# Small non-coding RNA deregulation in endometrial carcinogenesis

**DOI:** 10.18632/oncotarget.2911

**Published:** 2015-02-28

**Authors:** Maria Ravo, Angela Cordella, Antonio Rinaldi, Giuseppina Bruno, Elena Alexandrova, Pasquale Saggese, Giovanni Nassa, Giorgio Giurato, Roberta Tarallo, Giovanna Marchese, Francesca Rizzo, Claudia Stellato, Rossella Biancardi, Jacopo Troisi, Attilio Di Spiezio Sardo, Fulvio Zullo, Alessandro Weisz, Maurizio Guida

**Affiliations:** ^1^ Laboratory of Molecular Medicine and Genomics, Department of Medicine and Surgery, University of Salerno, Baronissi, Italy; ^2^ Fondazione IRCCS SDN, Napoli, Italy; ^3^ Genomix4Life Srl, Spin-Off of the Laboratory of Molecular Medicine and Genomics, University of Salerno, Baronissi, Italy; ^4^ Department of Medicine and Surgery and Division of Gynecology and Obstetrics, “SS. Giovanni di Dio e Ruggi d'Aragona - Schola Medica Salernitana”, University of Salerno Hospital, Salerno, Italy; ^5^ Department of Gynecology and Obstetrics and Pathophysiology of Human Reproduction, University of Naples “Federico II”, Napoli, Italy; ^6^ Division of Molecular Pathology and Medical Genomics, “SS. Giovanni di Dio e Ruggi d'Aragona - Schola Medica Salernitana”, University of Salerno Hospital, Salerno, Italy

**Keywords:** small non-coding RNA, endometrial cancer, miRNAs, piRNAs, snoRNAs

## Abstract

Small non-coding RNAs (sncRNAs) represent a heterogeneous group of <200nt-long transcripts comprising microRNAs, PIWI-interacting RNAs (piRNAs) and small-nucleolar-RNAs (snoRNAs) involved in physiological and pathological processes such as carcinogenesis and tumor progression. Aberrant sncRNA expression in cancer has been associated with specific clinical phenotypes, grading, staging, metastases development and resistance to therapy.

Aim of the present work is to study the role of sncRNAs in endometrial carcinogenesis. Changes in sncRNA expression were identified by high-throughput genomic analysis of paired normal, hyperplastic and cancerous endometrial tissues obtained by endometrial biopsies (*n* = 10). Using smallRNA sequencing and microarrays we identified significant differences in sncRNA expression pattern between normal, hyperplastic and neoplastic endometrium. This led to the definition of a sncRNA signature (129 microRNAs, 2 of which not previously described, 10 piRNAs and 3 snoRNAs) of neoplastic transformation. Functional bioinformatics analysis identified as downstream targets multiple signaling pathways potentially involved in the hyperplastic and neoplastic tissue responses, including Wnt/β-catenin, and ERK/MAPK and TGF-β-Signaling.

Considering the regulatory role of sncRNAs, this newly identified sncRNA signature is likely to reflect the events leading to endometrial cancer, which can be exploited to dissect the carcinogenic process including novel biomarkers for early and non-invasive diagnosis of these tumors.

## BACKGROUND

More than 80% of the human genome can be transcribed into RNA during homeostatic or disease-driven cellular responses. However, less than 2% of the human transcriptome is ultimately translated into proteins. The vast, untranslated fraction of the transcriptome includes a remarkable number of functional non-coding RNAs (ncRNAs) [1], comprising the so-called “small non-coding RNAs” (sncRNAs), made up of less than 200 nt. Four major groups of functional sncRNAs have been so far identified in mammals: micro- (miRNAs), PIWI-interacting- (piRNAs), small nucleolar- (snoRNAs) and endogenous small interfering- (endo-siRNAs) RNAs [2–5]. sncRNAs can exert large-scale and diverse effects on cellular processes by regulating gene expression, protein translation, and genomic organization. The best characterized sncRNAs so far are miRNAs (19–25 nt), known to be involved in post-transcriptional gene expression regulation by inducing mRNA degradation or translation inhibition. To date, more than 2000 human miRNAs have been identified, which are estimated to regulate more than 60% of coding genes [6]. Aberrant expression of miRNAs has been implied in numerous diseases, and miRNA-based therapies are in advanced stages of clinical trials or just starting entering clinic [7]. The sncRNA class defined as piRNAs have been linked to epigenetic and post-transcriptional gene silencing of retrotransposons and other genetic elements. They differ from miRNAs for size (26–31 nt vs. 19–25 nt), lack of sequence conservation and increased complexity [8]. Initial data indicated that PIWI proteins, with whom piRNA interact, had only germline-restricted functions but subsequent research expanded the role of these proteins in somatic tissues. Ectopic and over-expression of HIWIs (the human PIWI proteins) has been associated with several cancer types [9–12], and inhibition of HIWI expression can stop cancer cell growth [11]. Moreover, an emerging number of studies highlight the role of piRNAs or PIWI proteins in the regulation of tumorigenesis. Cheng et al. [13] demonstrated increased expression of piR-651 in gastric, colon, lung, and breast cancer tissues compared to normal adjacent tissues and down-regulation of piR-823 in gastric cancer tissues compared to normal tissues, suggesting its potential tumor suppressive role [13, 14]. Recently, Rizzo et al. [15] described cell cycle phase-specific piRNA regulation in regenerating liver, linked to regulation of growth-related genes, suggesting a role of these sncRNAs in growth control while Hashim et al. [16] showed evidence of an active piRNA pathway in breast cancer.

The last class of sncRNA, denominated snoRNAs, are involved in processing and modification of other RNAs, such as ribosomal and small nuclear spliceosomal RNAs. snoRNAs can be divided into two major structural classes, antisense box C/D and box H/ACA snoRNAs, based on the presence of short consensus sequence motifs. Most members of the two snoRNA classes guide the 2′-O-ribose methylations and pseudouridylations, respectively, of rRNA. snoRNAs have long been believed to modify and stabilize rRNAs for ribosome production. Several recent studies demonstrate that deregulation of snoRNAs is a common molecular event in lung carcinogenesis [17–19].

Endometrial cancer is the most frequent malignant tumor of the female reproductive tract and the fourth (by frequency) malignancy affecting the female population in developed countries [20, 21]. Type I endometrial cancer, which is the most frequent uterine malignancy (80% of all cases), consists of tumors of endometrioid histology. Shermann [22] proposed a mechanism by which indolent tumors develop from hyperplastic precursors in an estrogen-rich milieu. Hysteroscopy has an estimated sensitivity of 86.4% in the diagnosis of endometrial hyperplasia or adenocarcinomas [23]. However, hysteroscopic endometrial biopsy had limitations in as diagnostic approach for type I endometrial carcinoma: Peterson et al. [24] report that only 60% of grade I endometrial adenocarcinomas diagnoses based on this method was confirmed on final histopathology. Similarly, the intraoperative evaluation performed on frozen sections showed large discrepancies with the post-operative assessment made on the resected organ, especially in regard to lymphovascular invasion [25]. Altered miRNA expression has been found in endometrial adenocarcinoma [26], including up-regulation of the miR-200 family [27], of miR-205 and miR-210 [28] and miR-135a and miR-135b [29] and downregulaton of miR-133a and miR-133b [30]. Recently, miR-194 received much attention due to its potential prognostic role [31].

Expression of HIWI proteins has been reported in endometrial cancer [32] but the role of piRNAs has not been fully investigated during carcinogenesis [33, 34]. In addition, few data are available on the involvement of the other forms of sncRNA, such as snoRNAs, in this pathological process [35].

Next generation sequencing (NGS) provides a high-throughput method for exploring the diverse sncRNAs populations. This approach allows unbiased and accurate identification and quantitation of known and novel miRNAs and other sncRNAs, even when expressed at very low levels, or those exhibiting small differences in expression among samples, which cannot be detected by hybridization-based methods.

In the present study, coordinate analyses of sncRNAs and mRNAs expression were performed on samples of type I endometrial cancer biopsy coupled with samples of normal and hyperplastic endometrium obtained from the same patient, followed by functional analysis of the results. This represents an ideal model for the study of sncRNA role in the carcinogenesis process because it is characterized by the availability of healthy, hyperplastic and tumor tissue all taken from a single patient, allowing the comparative genetic analysis of normal and pathological tissues in the same genetic and hormonal background. In this condition it is possible to trace the changes in sncRNA expression occurring during the neoplastic transformation independently from other confounding factors to be taken into account when tissues are collected from different individuals.

The changes in sncRNA pattern in a patient is then generalized by means of comparison with changes observed in other patients, making the observations of general validity even if they are carried out in a relatively limited number of cases.

## RESULTS AND DISCUSSION

### Characterization of small non-coding RNAs deregulation in tumoral, hyperplastic and normal-adjacent endometrial tissues

Genome-wide identification of sncRNAs expressed in a given tissue type in both normal and pathologic conditions allows the study of the complex small RNA-mediated regulatory networks and of the modifications imposed by the carcinogenic process. The involvement miRNAs in carcinogenesis has been under investigation since the discovery of distinct expression patterns among different tissues and that their expression changes significantly in cancer tissues compared to normal ones [36]. Although miRNAs are the most studied sncRNAs, the importance of other sncRNAs, in particular snoRNAs and piRNAs is increasingly being recognized but little is known about their deregulation and involvement in the carcinogenesis process. Indeed, although several studies focusing on alterations of miRNA expression in endometrial cancer are available [37, 38], genome-wide expression profiling data relative to other sncRNAs in endometrial adenocarcinoma are still missing. Identification of differentially expressed sncRNAs between normal, hyperplastic and tumor tissue is a crucial point for a comprehensive characterization of the carcinogenesis process. We analyzed sncRNAs by next-generation sequencing in type I endometrial cancer samples to evaluate their possible deregulation during carcinogenesis. To this aim, ten patients (indicated as 1, 3, 4, 5, 6, 7, 14, 15, 16 & 17) and 3 healthy controls (indicated as 2, 8 & 9) were selected, following initial characterization according to defined clinico-pathological parameters (Table 1 and Supplementary Figure S1B). For each individual, three endometrial biopsies were obtained, when possible, from tumor, hyperplastic and adjacent normal tissue, by means of hysteroscopy. In total we collected ten normal (1N, 2N, 3N, 4N, 5N, 7N, 8N, 9N, 14N, 15N), six hyperplastic (1H, 3H, 4H, 5H, 6H, 7H) and ten tumor tissue samples (1T, 3T, 4T, 5T, 6T, 7T, 14T, 15T, 16T, 17T). Normal samples were collected the farthest possible from the area presenting a hyperplastic or cancerous lesion, to reduce the possibility of cross-contaminations.

**Table 1:** Characteristics of patients and healthy controls of normal, hyperplastic and neoplastic tissues analyzed

Characteristics	N°	Patients
Age		
>55	7	2,3,6,7,14,15,16
≤55	6	1,4,5,8,9,17
Tissue Categories		
Healthy Control	3	2N,8N,9N
Normal	7	1N,3N,4N,5N,7N,14N,15N
Atypical Hyperplasia	6	1H,3H,4H,5H,6H,7H
Endometrioid	10	1T,3T,4T,5T,6T,7T,14T,15T,16T,17T
Stage (FIGO)		
I–II	9	1T,3T,4T,5T,7T,14T,15T,16T,17T
III–IV	1	6T
Histological grade		
G1	5	1T,4T,14T,15T,16T
G2	3	3T,7T,17T
G3	2	5T,6T
Lymph node metastasis		
Positive	1	6T
Negative	4	3T,4T,5T,15T
Nx	5	1T,7T,14T,16T,17T
Stage (TNM)		
T1 and T2	9	1T,3T,4T,5T,7T,14T,15T,16T,17T
T3 and T4	1	6T

**Figure 1:**
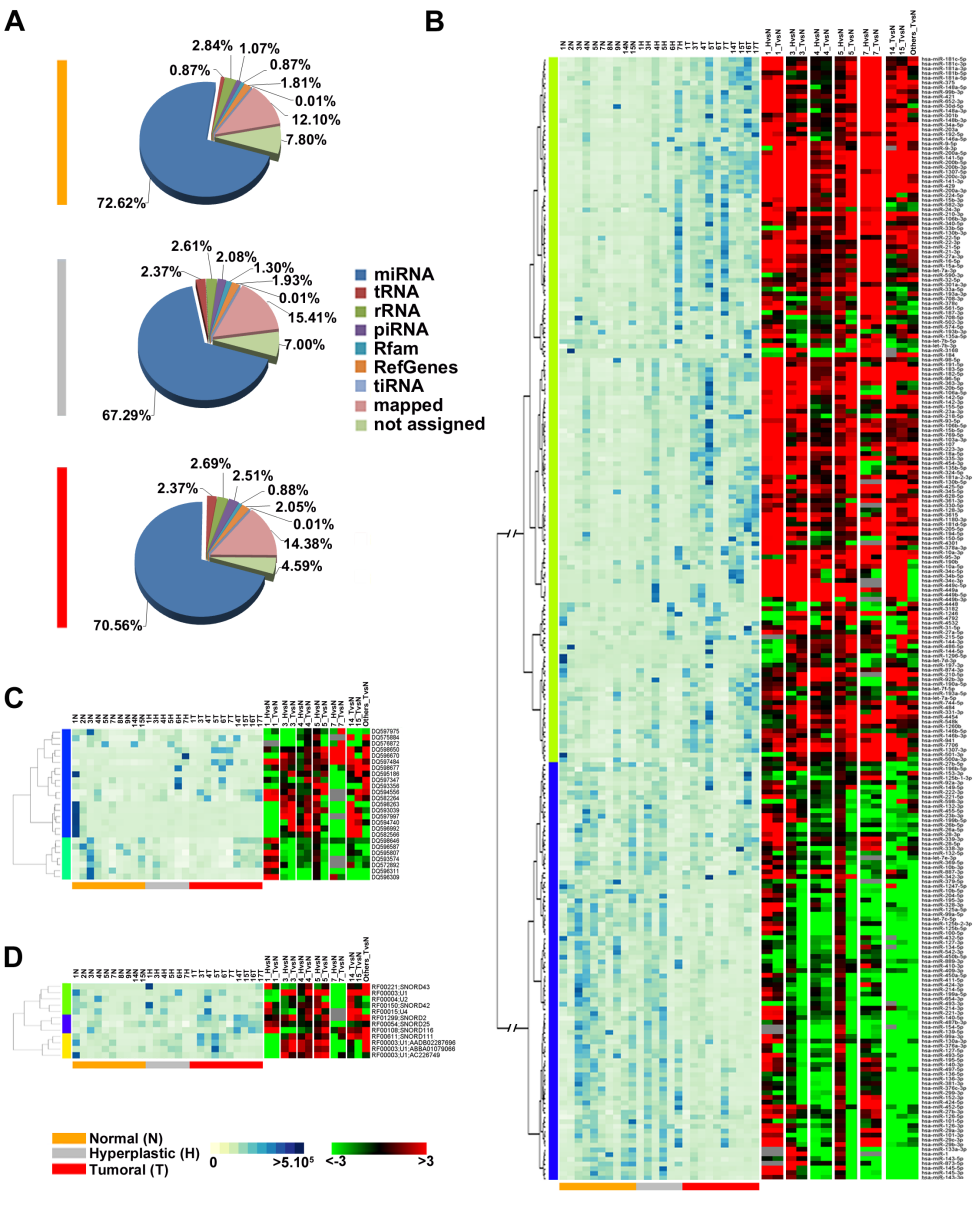
Changes in sncRNAs expression during endometrial carcinogenesis **(A)** Pie-charts showing the percentage of small non-coding RNA species identified in each tissue type by smallRNA-Seq. Heatmaps showing expression levels (RPM, left) with respect to the fold-change (FC) (right) for miRNAs **(B)**, piRNAs **(C)** and snoRNAs **(D)** differentially expressed during carcinogenesis. Only sncRNAs with base mean read count ≥30 in at least 3 samples and with significant fold-change in at least 50% of patients (|FC| ≥1.5, *p*-value 0.05) were showed. Expression levels are displayed from white (low expression) to dark blue (high expression) while differentially expressed sncRNAs are displayed from green (under-expressed) to red (over-expressed). RNAs not differentially expressed in a given sample are in black; grey indicates missing data.

More than 300 million sequences were obtained by smallRNA-Seq analysis of the 26 samples sequenced, after filtering out low quality reads and trimming the adaptor, with an average of 10 million reads/sample. In all cases we observed a bimodal distribution of read length, with a major size class peaking at 22nt, corresponding to the length of miRNAs, and a smaller one at 28–34 nt, a length compatible with other sncRNAs such as piRNAs. The sequences corresponding to known miRNAs (present in miRBase Release 19) had a similar abundance independently from the tissue, ranging from 67% for the hyperplastic to 72% for the tumor samples (Figure 1A). About 2% of reads aligned to known piRNA sequences reported in piRNABank, in agreement with previous reports in other somatic tissues [15, 39], and 2% of sequences aligned to Rfam database entries. Overall, we identified 1,268 sncRNAs (1,019 miRNAs, 145 piRNAs and 104 snoRNAs), among which 448 (359 miRNAs, 62 piRNAs and 27 snoRNAs) were found to be expressed at significant level (showing > 10 RPM in at least three samples). Furthermore, 60%, 66%, 53% of the total miRNAs, piRNAs and snoRNAs repertoire, respectively, was identified by detection of 10 RPM in at least one sample. Interestingly, several piRNAs, such as hsa_piR_020829_DQ598677, hsa_piR_019914_DQ597347 and hsa_piR_016735_DQ593039, displayed a very high level of expression (Mean RPM ≥ 4,500). The full list of identified sncRNAs is available in Supplementary Table S1.

To investigate whether miRNA, piRNA and snoRNA expression patterns changes during endometrial carcinogenesis, differential expression analysis was performed and a pool of sncRNAs specifically deregulated in hyperplastic and tumor tissue was identified in each patient. The results were then combined to define common patterns of sncRNAs deregulation. We identified 276 sncRNAs (239 miRNAs, 25 piRNAs and 12 snoRNAs: Figure 1B, and 1D respectively) differentially expressed in at least 50% of the patients, considering |FC| ≥1.5, *p*-value 0.05 and base mean read count ≥30. The full list of differentially expressed sncRNAs is available in Supplementary Table S2.

This dataset represent, to our knowledge, the first comprehensive sncRNA profile in endometrial carcinogenesis, including less characterized classes of sncRNAS that are emerging as new players in cancer and could play important roles in carcinogenesis and tumor progression [13, 19, 40].

It is worth noting that the miRNA data reported here share many similarities with those previously characterized with different analytical approaches [38, 41–44].

Interestingly, in hyperplastic and neoplastic tissues we observed the over-expression of members of the miR-200 family (has-miR-141, has-miR-200a, has-miR-200b, has-miR-200c, has-miR-141, has-miR-429), which is known to be involved in epithelial-to-mesenchymal transition (EMT) [41].

### Identification of candidate novel miRNAs

**Figure 2:**
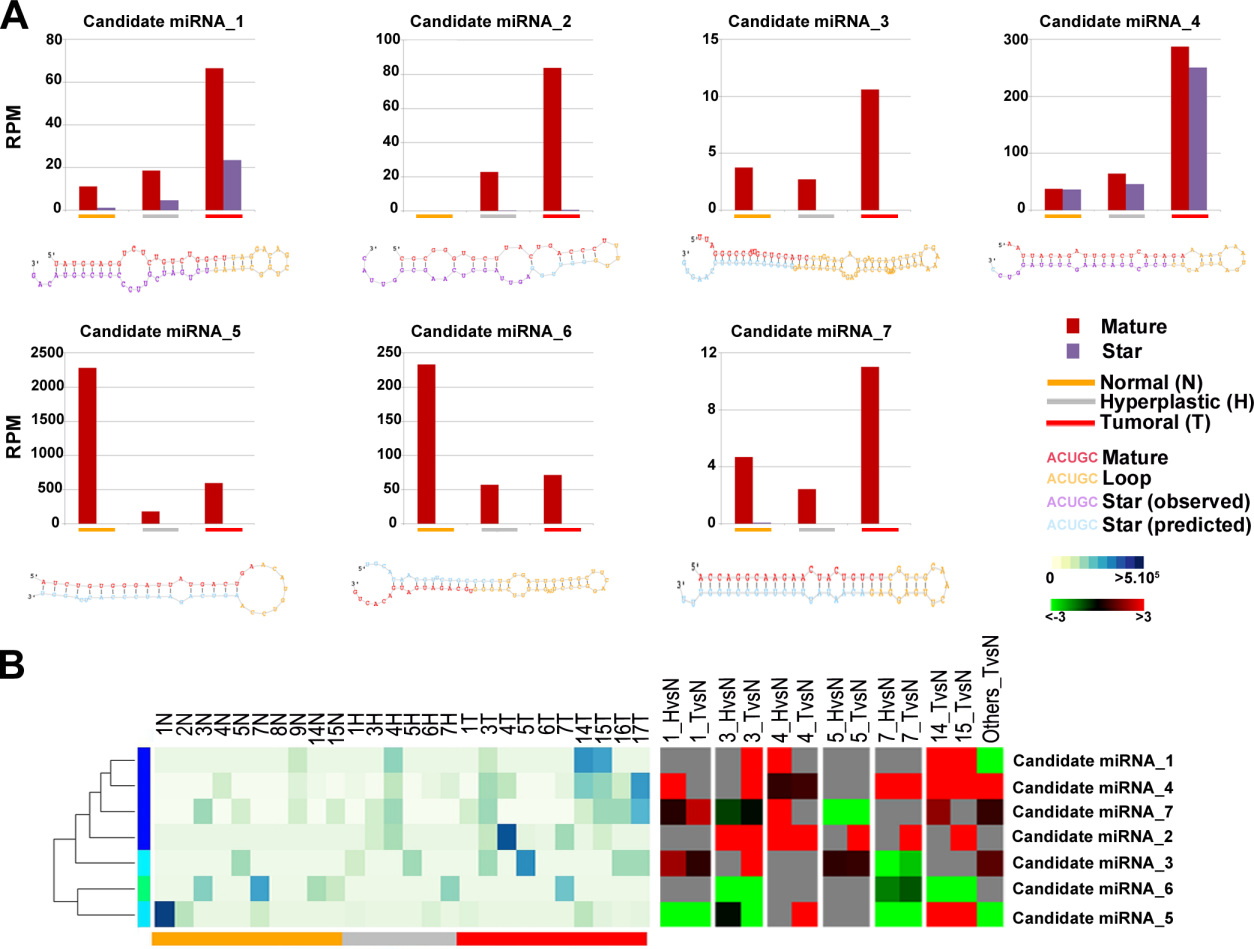
Novel miRNA candidates discovered by next generation sequencing **(A)** Number of reads found for each tissue type and secondary structures of the novel miRNAs. Histograms show the total number of reads sequenced for each histotype (normal: orange; hyperplastic: grey; tumor: red) and for each portion of the miRNA identified (mature: blue, loop: red and star: green). All novel miRNAs were computationally predicted to form stem-loop hairpin structures but each novel miRNA varied in the location, number and size of bulges. Putative secondary structures for the seven novel miRNAs discovered in this study are shown below the corresponding histograms. Red, yellow, blue and purple indicate respectively the mature sequence, the loop structure, the predicted star sequence and the star sequence when identified in our sequencing data. **(B)** Heatmaps showing the expression level (RPM) of 7 novel miRNAs (left) and the relative fold-difference for each patient (right). Expression intensities are displayed from white (low expression) to dark blue (high expression) while over-expressed miRNAs are indicated in red and under-expressend miRNAs are indicated in green; grey indicates missing data.

Since smallRNA-Seq allows to discover novel sncRNAs still not annotated in public databases, including those occurring at low frequencies [45], sequences not mapping to known mature miRNAs were analyzed with iMir tool (see Materials and Methods) to predict potentially novel miRNAs that could be deregulated in the carcinogenesis process. The reads that aligned against tRNAs or mRNAs (from UCSC Genome Browser), rRNAs or piRNAs (from Nucleotide NCBI database) and other known sncRNAs (from RFam datasets) were excluded prior to the analysis in order to reduce false-positive rate in predicting novel miRNAs. The remaining reads were analyzed by using miRDeep2 algorithm [45], which is able to identify novel miRNAs with high accuracy [45, 46]. Potential precursor sequences were then searched *in silico* and tested for their ability to form a characteristic hairpin; the resulting sequences were further analyzed to determine features such as thermodynamics stability, presence of star sequences and phylogenetic conservation [46]. Using these criteria, seven novel miRNAs were identified (Figure 2A, 2B left panel and Supplementary Table S3), all expressed with more than 30 reads in at least three samples, and, for two of them (candidate miRNA_1 and candidate miRNA_4, Figure 2A), with evidence of expression also of the corresponding star sequences. The predicted precursor structure and the sequence of miRNA star corresponding to the novel miRNAs are showed in Figure 2A. It is worth noting that the seed regions of 6 novel miRNAs matched with miRNAs of other species (one mismatch) and are broadly conserved among vertebrates, particularly mammals. Moreover, the seed sequences of candidate miRNA_2 and candidate miRNA_3 match those of human miR-886–3p family and miR-1296 family, respectively. All these evidences strongly support the possibility that these small RNAs are authentic endometrial miRNAs. Interestingly, differential expression analysis of these novel miRNA candidates shows a significantly different expression in hyperplasia and/or tumor tissues in at least three patients (|FC| ≥ 1.5 and *p*-value ≤ 0.05, Figure 2B right panel and Supplementary Table S3).

### Identification of sncRNA expression signatures in endometrial carcinogenesis

**Figure 3:**
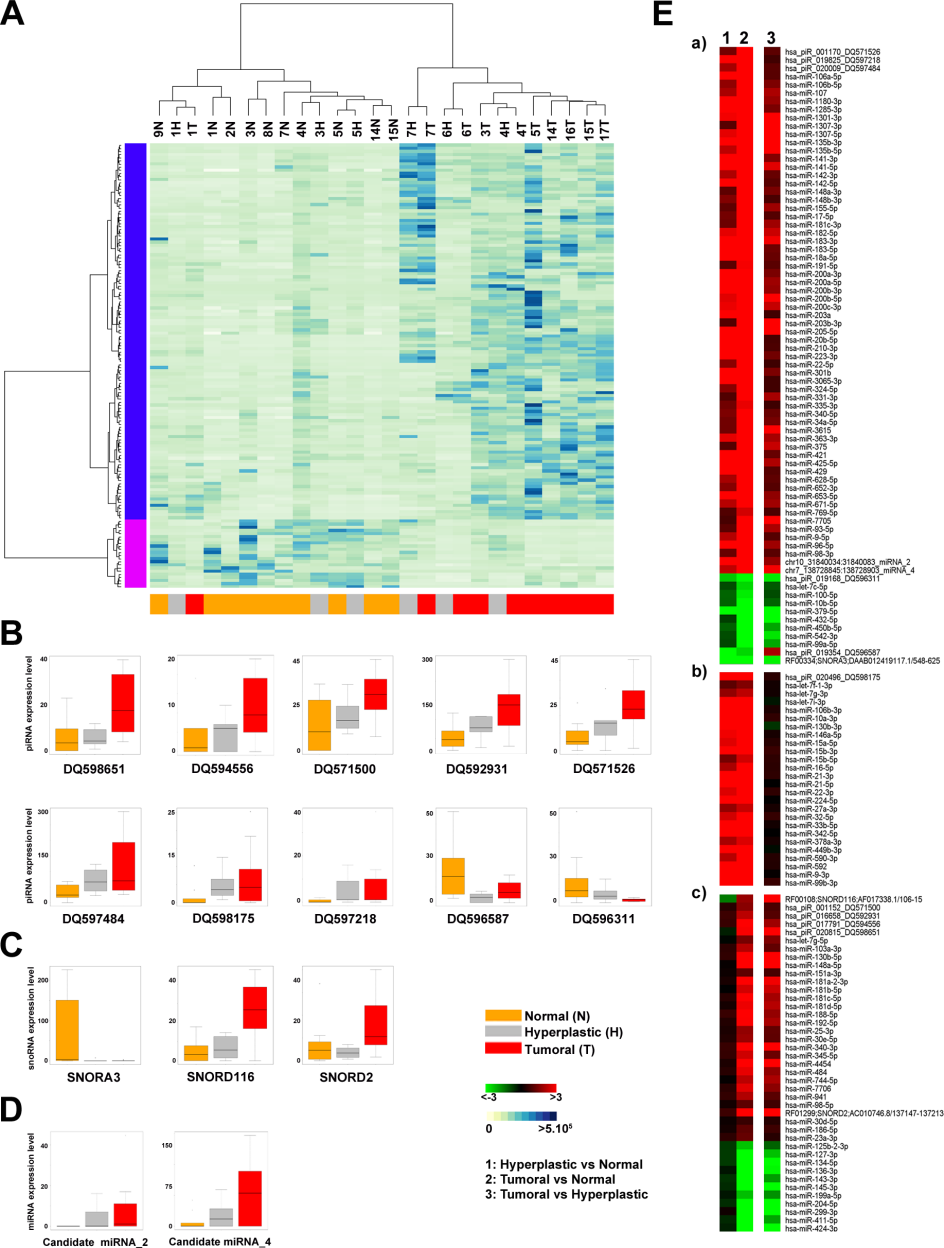
Identification of sncRNAs signature discriminating between normal and tumor samples **(A)** Unsupervised hierarchical clustering of samples based on the expression level (RPM) of 142 sncRNAs (127 known miRNAs, 2 new miRNAs, 10 piRNAs and 3 snoRNAs) discriminating among normal (yellow), hyperplastic (grey) and tumor (red) samples (Kruskal-Wallis Test, *p*-Value <0.05). Each row corresponds to one *sncRNA*. BoxPlots showing the expression level (RPM) of 10 piRNAs **(B)**, 3 snoRNAs **(C)** and 2 new miRNAs **(D)** identified in the signature. (E) Heatmaps showing fold difference values (ratio of mean RPM for each tissue: 1 = hyperplastic *vs* normal, 2 = tumor *vs* normal and 3 = tumor *vs* hyperplastic) of the sncRNAs identified in the signature. The sncRNAs are grouped as follows: (a) 83 showing a similar pattern of expression (b) 26 differentially expressed in both the hyperplastic and tumor compared to adjacent normal tissue, but not changing between hyperplastic and tumor tissues (c) 28 showing different trends.

In order to recognize differential sncRNA expression profile among the three different tissue types, we applied the Kruskal–Wallis (one-way analysis of variance) test on the identified sncRNA expression profiles, including also the 7 novel miRNAs. This led to the definition of a sncRNA signature comprising 129 miRNAs, 2 of which not previously described, 10 piRNAs and 3 snoRNAs, whose expression level (RPM, Supplementary Table S1) discriminates tumour from normal endometrial tissues. Indeed, hierarchical clustering analysis (Figure 3A) shows a clear separation of cancers and normal tissues, with the exception of samples T1, while hyperplasia result equally distributed between the normal and tumor clusters. The fact that some hyperplastic samples appear more closely related to cancer while other to normal endometrium could relate to heterogeneity of these lesions, due to the existence of different degrees of atypical hyperplasia, undistinguishable with the standard pathological tests available.

The box plots in Figure 3 show the behaviour of each piRNA (hsa_piR_001152_DQ571500, hsa_piR_001170_DQ571526, hsa_piR_016658_DQ59293, hsa_piR_017791_DQ594556, hsa_piR_019168_DQ596311, hsa_piR_019354_DQ596587, hsa_piR_019825_DQ597218, hsa_piR_020009_DQ597484, hsa_piR_020496_DQ598175, hsa_piR_020815_DQ598651, Figure 3B), snoRNA (RF00334;SNORA3;DAAB012419117.1/548–625, RF00108;SNORD116;AF017338.1/106–15, RF01299;SNORD2;AC010746.8/137147–137213, Figure 3C) and new miRNA (candidate miRNA_2 and _4, Figure 3D) in the different sample types. The corresponding *p*-values are reported in Supplementary Table S4.

Furthermore, differential expression analysis performed on paired sample groups identifies 142 differentially expressed sncRNAs (|FC| ≥ 1.5) (Figure 3E and Supplementary Table S4). In particular, 19 miRNAs, 2 piRNAs and 1 snoRNA result under-expressed and 110 miRNAs (2 new and 108 known), 8 piRNAs and 2 snoRNAs over-expressed in cancerous respect to normal tissues. We also observed some specific trends, shown in Figure 3E and summarized as follows: a) 75 sncRNAs over- or under-expressed in both hyperplasia and tumors (Figure 3E-a), but more so in the latter respect to the former; b) 28 sncRNAs showing similar differences in the two pathological tissues respect to normal endometrium (Figure 3E-b); c) 41 sncRNAs changing significantly only in cancer (Figure 3E-c).

### Functional analyses of the cellular pathways affected by sncRNA dysregulation in endometrial carcinogenesis

As miRNAs modulate posttranscriptionally the targeted mRNAs by binding directly to their 3′-UTR and promoting mRNA degradation or translation inhibition, an increase in the intracellular concentration of a given miRNA may result in reduced levels of the proteins encoded by its target mRNAs. In the case of piRNA, while their main function in the germline is to silence transposons both transcriptionally and post-transcriptionally [47, 48], their roles in somatic cells is still unknown. Recently it has been shown that these small RNAs can also bind to mRNAs and thereby control their stability and translation efficiency [49, 50], similarly to miRNAs. Based on this evidence, we postulate that the changes in piRNA expression occurring in endometrial carcinogenesis may affect resident mRNA activity and influence downstream endometrial cell functions. To search for mRNA targets of both known and novel sncRNAS comprising the endometrial cancer ‘signature’ identified here, a gene expression profiling was generated by cDNA microarrays, focusing on the biopsies from the 5 patients for whom the three histotypes were available. An equimolar RNA pool was generated in each case, named Normal (N), Hyperplastic (H) or Tumoral (T) and used for cRNA synthesis and microarray hybridization. Overall, considering |FC|≥ 2.0 and *p*-value 0.001 we identified 618 transcripts (Supplementary Figure S2) which were differentially expressed in at least one paired comparison (hyperplastic *vs* normal, cancer *vs* normal and/or 3: cancer *vs* hyperplastic), out of about 18,500 RNAs detected (detection *p*-value ≤ 0.05). Among them, 302 showed changes in the hyperplastic tissue (203 over- and 99 under-expressed) and 452 the tumor tissue (164 over- and 288 under-expressed) with respect to normal endometrium of the same patients. Interestingly, down-regulated mRNAs where the majority in tumor (63%) compared to hyperplastic (33%) tissues, consistent with the greater percentage of over-expressed miRNAs in the former. Also in this case, it was possible to observe different trends (Supplementary Figure S2) summarized as follows: a) 205 mRNAs show a similar change, but at different levels, in all the three cases considered (over- or under-expressed in all three comparisons, Supplementary Figure S2A); b) 177 mRNAs are differentially expressed both in the hyperplastic and in tumor tissue, but without significant differences in the two pathological tissues (over- or under-expressed only in comparisons 1 and 2, Supplementary Figure S2B); c) 17 mRNAs show opposite behavior, respect to normal endometrium, in hyperplasia compared to tumor (Supplementary Figure S2C); d) 219 mRNAs showing different expression patterns, including 65 mRNAs differentially expressed only in the tumors and 35 only in hyperplasia (Supplementary Figure S2D). The full list of these transcripts, with relevant information, is available in Supplementary Table S5.

A functional analysis of transcripts whose level was altered in endometrial cancer reveals that in many cases the proteins they encode are involved in cellular processes characteristic of transformed cells, including in particular cell proliferation, survival and differentiation and cell motility, migration and adhesion. This result is in line with previous observations with similar analyses in endometrial cancer [51–53].

Interestingly, transcripts for PEG3 (paternally expressed 3, PW1) and SOCS2 (suppressor of cytokine signaling 2, STATI2) were down-regulated, while HOXB7 and HOXB6 mRNAs resulted up-regulated in both the hyperplastic and neoplastic stages. PEG3 is known to be underexpressed in gliomas, as well as in ovarian, breast and other gynecologic cancers [54–57]. The precise function of the encoded protein is not clear, but several evidences suggest that it may play a role in the p53/c-myc-mediated apoptosis pathway and in Wnt signaling pathway by binding to beta-catenin and promoting its degradation via a p53/Siah1-dependent and GSK3beta-independent proteasomal pathway. This suggests that PEG3 could function as tumor suppressor [58, 59]. Also SOCS2 is a tumor suppressor involved in the JAK/Stat signaling pathway, whose deregulation is associated with malignant progression [60, 61]. It has been shown that experimental over-expression of SOCS proteins in cancer cells reduces STAT activity, inhibits proliferation and induces apoptosis [61–64]. The HOX genes comprise a family of homeodomain-containing transcription factors that determine cell identity during development. Aberrant HOX genes expression has been reported to mediate oncogenesis in several human cancers, including hepatocellular [65] and ovarian cancer [66], as well as in acute myeloid leukemia (AML) [67]. These genes play a key role in the inhibition of apoptosis, DNA repair and enhanced cell motility [68].

To explore the biological and functional role exerted by the aberrantly expressed sncRNA of the ‘signature’ discriminating cancerous from normal endometrial tissues, their downstream mRNA targets were searched *in silico* using different computational approaches. To evaluate their involvement in biological networks, the results were then combined with the gene expression profiling data using Ingenuity Pathway Analysis (IPA) tools, as described in Materials and Methods. Putative targets of the two newly identified miRNAs were searched for with TargetScanHuman [Release5.2, 69]. This analysis led to the identification of 526 endometrial mRNA targets, including 65 differentially expressed (|FC| ≥ 1.5 and *p*-value 0.001) in hyperplastic and/or tumor tissues. Several cellular processes and canonical pathways were found to be potentially affected by these miRNAs, such as cellular development, growth and proliferation, cell cycle (G1/S checkpoint), cellular movement and cell death and survival, TGF-β, ERK/MAPKand Wnt/β-catenin signaling pathways (Supplementary Table S6).

The search for the putative targets of the piRNA identified in our study was pursued applying a method based on sequence complementarity that allows the prediction of biologically relevant RNA-RNA interactions [15, 49] (Figure 3). By applying stringent thermodynamic parameters for RNA-RNA binding, each piRNA analyzed was found to be complementary to a number of mRNAs ranging from 28 to 308. In total, 1,526 putative mRNA targets were found among those significantly expressed in our samples, among which 170 resulted to be differentially expressed (|FC| ≥ 1.5 and *p*-value 0.001) in hyperplastic and/or tumor tissues. As for miRNA-targeted mRNAS, the proteins encoded by the mRNAs putatively targeted by piRNA are involved in key cellular events highly related to the carcinogenetic process, such as cell death and survival, growth and proliferation, cell to cell signaling, cell cycle and DNA replication (Supplementary Table S7), while 38 of them have been already previously associated to endometrial cancer (IPA knowledge database 2014).

Finally, the known miRNA targets were searched with the microRNA Target Filter database of Ingenuity suite using experimentally validated interactions from TarBase and miRecords, as well as highly predicted miRNA-mRNA interactions from TargetScan. The 127 miRNAs of interest were grouped in 79 miRNA families (sharing the same seed region) that, combined, can potentially target 8,418 endometrial mRNAs, including 909 differentially expressed (|FC| ≥ 1.5 and *p*-value 0.001) in hyperplastic and/or tumor tissues, 60% of which showed FC values inversely correlated with those of the related miRNA (Supplementary Table S8). Among several over-represented pathways, this analysis confirmed the potential effect of these sncRNAs on cell cycle regulation and ERK/MAPK, JAK/Stat and Wnt/β-catenin signaling pathways (Supplementary Table S9). Concerning the last pathway, it is interesting that 6 over-expressed sncRNAs (piR_020496_DQ598175, Candidate miR-4, miR-103–3p, miR-155–5p, miR-16–5p and miR-17–5p can all target the down-regulated TLE4 (transducin-like enhancer of split 4), a transcriptional corepressor that binds to a number of transcription factors in this cascade.

Interestingly, 9 over-expressed miRNA families (miR-186–5p, miR-205–5p, miR-181a-5p, miR-9–5p, miR-141–3p, miR-155–5p and miR-135a-5p) target the down-regulated PEG3, which results strongly repressed in both hyperplastic and tumor tissues (see above). This finding suggests a possible role for down-regulation of this oncosuppressor in endometrioid cancers.

Concerning the aforementioned down-regulated SOCS1 and SOCS2, we found that the first is targeted by miR-22–3p and hsa-miR-155–5p [miRNAs w/seed UAAUGCU, experimentally observed target, 70], while the second is a target of miR-30e-5p, all over-expressed in our tumoral samples.

As a further example of the information provided by the present data, we found that the up regulated miR-181a-5p, miR-27a-3p and hsa-miR-107, miR-103–3p, hsa-miR-200a-3p, miR-141–3p, miR-203–3p, miR-27a-3p, miR-425–5p, miR-96–5p all target the down-regulated SEMA3B and SEMA6A mRNAs. Interestingly, these are encoded by two genes belonging to the axon-guidance family, that plays a critical role in the guidance of growth cones during neuronal development, and both have been shown to be inactivated in human cancers, where they have been proposed to act as tumor suppressor genes by inducing apoptosis [71].

**Figure 4:**
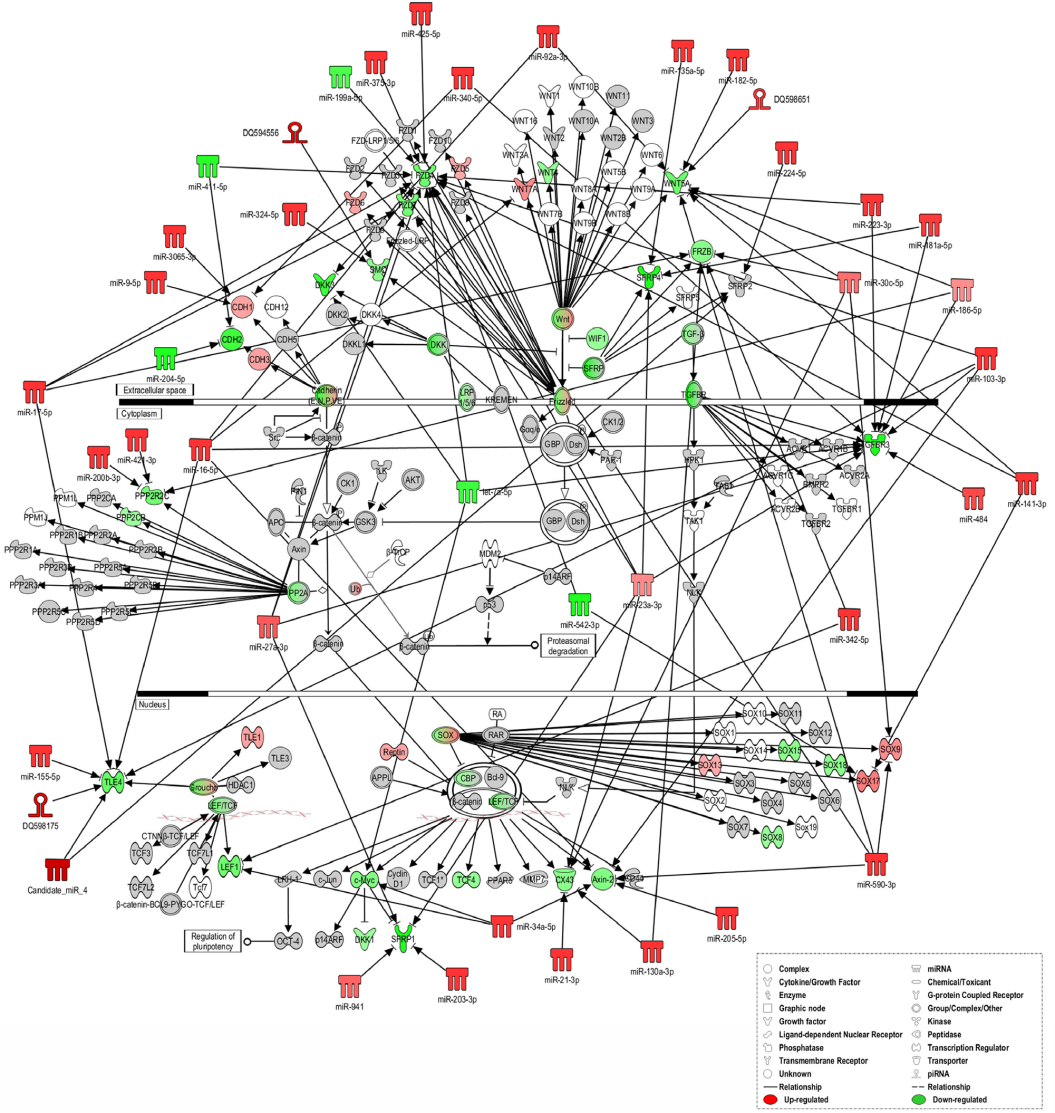
Ingenuity pathways analysis of Wnt/β-catenin Signaling IPA of “sncRNA signature” and downstream targets involved in Wnt/β-catenin Signaling in tumor *versus* normal tissues. sncRNAs and mRNAs with significant increases in expression are shown in red, sncRNAs and mRNAs with significant decreases are shown in green, and which that were identified but did not change significantly are shaded in gray. Color intensity reflects the level of expression. Types of molecules are annotated in the legend in the box.

These data reveal that miRNAs (known and novel) and piRNAs can exert a synergic modulation of several signaling pathways, such as ERK/MAPK, TGF-β and Wnt/β-catenin, whose activation is already occurring in the hyperplastic lesion but becomes more pronounced in the neoplastic tissue (Figure 4 and Supplementary Figure S3 A-E).

In conclusion, our sequencing analysis has identified a novel sncRNA signature encompassing different sub-classes of deregulated small RNAs, differentiating normal form hyperplastic and tumoral endometrium. This large dataset can provide key information on the molecular mechanisms of carcinogenesis. Furthermore, it can be exploited to identify biomarkers that may assist in the early and non-invasive diagnosis of human endometrial cancers.

## MATERIALS AND METHODS

### Patients and tissue collection

Patients were subjected to transvaginal ultrasound and office hysteroscopy in the Departments of Obstetrics and Gynecology of the University of Salerno (Salerno, Italy) and University of Naples Federico II (Naples, Italy). Endometrial biopsies from tumor (T), adjacent normal (N) and hyperplastic (H) tissue specimens (when available) from 10 patients and Normal endometrial tissue from 3 healthy donors were selected for this study (Supplementary Figure S1A). As healthy donors, patients who underwent hysteroscopy for abnormal uterine bleeding (AUB) non related to cancer pathology were recruited.

Median age was 58 year old (range 32–84). 9 patients had stage I–II disease and 1 had stage III disease. 8 cases were G1-G2 and 2 were G3-G4. See Table 1 and Supplementary Figure S1B for more detailed clinical characteristics of patients.

The tissues were kept on dry ice at all times during tissue sampling and handling. The histology of each tumor was classified according to World Health Organization criteria, whereas surgical staging followed International Federation of Gynecology and Obstetrics (FIGO) standards (FIGO 2009). The ethics committee of Azienda Ospedaliera Ospedali Riuniti S. Giovanni e Ruggi d'Aragona di Salerno approved this study and informed consent was obtained from all patients.

### RNA isolation and quality controls

Tissue specimens samples were distrupted and homogenaized using TissueLyser LT (Qiagen). Total RNA was extracted with miRVana RNA isolatin Kit (Ambion) according manufacturers protocol. Before use, RNA concentration in each sample was assayed with a ND-1000 spectrophotometer (NanoDrop) and its quality assessed with the Agilent 2100 Bioanalyzer with Agilent RNA 6000 nano kit (Agilent Technologies).

### Small RNA sequencing and data analysis

1 μg of total RNA was used in a library preparation according to the Illumina TruSeq small RNA sample preparation protocol (Illumina, USA). Sized smallRNA libraries were gel purified and sequenced on HiSeq 1500 (Illumina, USA) at a concentration of 10pM for 50 plus 7 additional cycles for indexes sequencing. Each library was sequenced in three technical replicate.

The data obtained from the sequencer were analyzed using iMir, a modular pipeline for comprehensive analysis of small RNA-Seq data, comprising specific tools for adapter trimming, quality filtering, identification of known sncRNAs, novel miRNA prediction, differential expression analysis and target prediction [72, 73].

Reads from technical replicates were summed and normalized using reads per million method (RPM). To minimize spurious detection due to sequencing errors sncRNAs have been considered as expressed when they were detected by at least ≥ 10 RPM reads (75th percentile of RPM values) per sncRNA, corresponding approximately to 100 reads for an average library.

Heatmaps showing the expression level (RPM) were generated with R software after performing one-way hierarchical clustering of sncRNAs with the Kendall rank correlation.

Identification of differentially expressed sncRNAs was performed with the Bioconductor DESeq package available in iMir tool [72, 73]. A negative binomial distribution model was used to test differential expression in deep sequencing datasets. Data were filtered according to read count value (threshold: 10 reads) and those showing fold-change ≤−1.5 or ≥1.5 (|FC| ≥1.5), with a *p*-value ≤ 0.05 were considered as differentially expressed. The reads of the 3 patients from whom we do not have the normal tissue but only the tumoral one (6T, 16T & 17T) were pooled and compared with the read pool of the 3 healthy controls (2N, 8N & 9N) and indicated in the figures as Others. Heatmaps showing the fold-change of differentially expressed sncRNAs were generated with the Multiexperiment Viewer 4.9 software (TM4).

The RPM data were statistically analyzed using the Kruskal–Wallis test (*p*-value ≤0.01). Two-way cluster analysis was carried out using R software for Kendall rank correlation.

Detection of other sncRNAs and novel miRNAs prediction was performed with Module 3 of the iMir tool (miRanalyzer and miRDeep2) [72].

Putative piRNA target RNAs were identified by sequence complementarity between each piRNA (piRNABank, Institute of Bioinformatics and Applied Biotechnology, Bangalore, India) and the 5′-UTRs, CDSs or 3′-UTRs of all known human RNAs (RefSeq gene annotations), using miRanda v3.3a [74], an algorithm for detection of potential small RNA binding sites in RNAs, applying stringent alignment score (sc; ≥170) and energy threshold (en; ≤−20.0 kcal/mol). The functional analyses were generated through the use of IPA (Ingenuity Systems, http://www.ingenuity.com) to identify the biological processes that are significantly associated (*p*-value ≤0.05) with mRNAs targeted by piRNAs, as previously described [15].

Raw small non-coding RNA sequencing data have been deposited, in a format complying with the Minimum Information about a high-throughput SeQuencing Experiment (MINSEQE) guidelines in the EBI ArrayExpress database (http://www.ebi.ac.uk/arrayexpress) with Accession Number E-MTAB-2795.

### Microarray and data analysis

3 RNA pool were generated for each tissue type: Normal (samples: 1N, 3N, 4N, 5N, 7N), Hyperplastic (samples: 1H, 3H, 4H, 5H, 7H) and Tumoral (samples: 1T, 3T, 4T, 5T, 7T). Gene expression profiling was performed using 500 ng of each RNA Pool as described before [73]. For each Pool, 750 ng of cRNA were hybridized for 18 hrs at 55°C on Illumina HumanHT-12 v4.0 BeadChips (Illumina Inc.), according to the manufacturer's instructions and subsequently scanned with the Illumina iSCAN. Data analyses were performed with GenomeStudio software version 2011.1 (Illumina Inc.). Data was normalized with the quantile algorithm and genes were considered as detected if the detection *p*-value was lower than 0.01. Statistical significance was calculated with the Illumina DiffScore, a proprietary algorithm that uses the bead standard deviation to build an error model. Only genes with a DiffScore ≤−30 and ≥ 30, corresponding to a *p*-value of 0.001, were considered as statistically significant.

Heat Maps were generated with the Multiexperiment Viewer 4.9 software (TM4) after performing one way hierarchical clustering of transcripts with the average linkage method and Euclidian distance.

Raw microarray data have been deposited, in a format complying with the Minimum Information About a Microarray Gene Experiment (MIAME) guidelines of the Microarray Gene Expression Data Society (MGED), in the EBI ArrayExpress database (http://www.ebi.ac.uk/arrayexpress) with Accession Number E-MTAB-2796.

### Enriched pathways and bibliographic networks discovery

miRNA target prediction was performed using Ingenuity Pathway Analysis (IPA, Ingenuity Systems). The dataset was mined for significant pathways with the IPA library of canonical pathways, and networks were generated by using IPA as graphical representation of the molecular relationships between genes and gene products. The significance of the association between the list of differentially expressed genes, miRNAs and the Canonical Pathway was measured using a Fisher's exact test to calculate a *p*-value (*p* ≤ 0.05). Fisher's exact test results were also corrected for multiple testing using FDR.

## SUPPLEMENTARY FIGURES




















