# Supplementary material for: Small non-coding RNA deregulation in endometrial carcinogenesis
Source: Oncotarget. 2015 Feb 28;6(7):4677–91. doi: 10.18632/oncotarget.2911 (PMC4467107; doi:10.18632/oncotarget.2911)
Supplement: Supplementary file 1 [file oncotarget-06-4677-s001.pdf]

SUPPLEMENTARY FIGURES

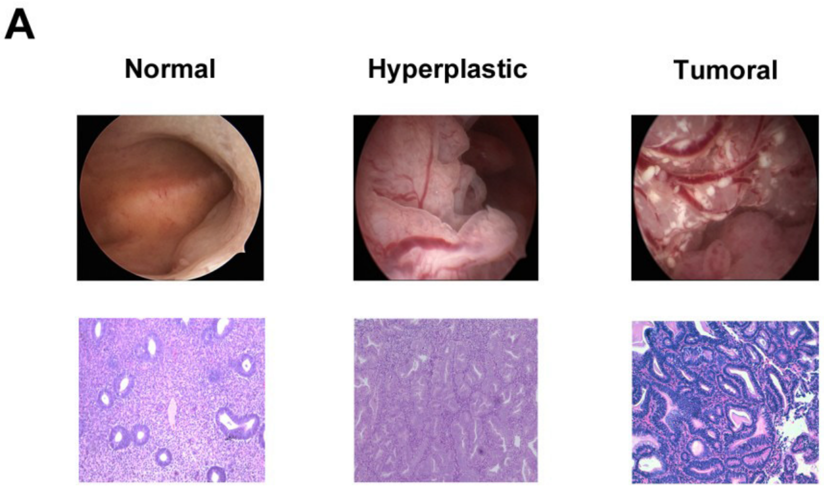

B

Patients	Tumor IST	pT	pN	G	S	Age
1	Endometrioid	T1a	NX	G1	IA	32
2						73
3	Endometrioid	T1	N0	G2	I	59
4	Endometrioid	T1	N0	G1	I	55
5	Endometrioid	T1a	N0	G3	IA	54
6	Endometrioid	T3a	N1	G3	IIIC1	84
7	Endometrioid	T1a	NX	G2	IA	73
8						55
9						37
14	Endometrioid	T1a	NX	G1	IA	58
15	Endometrioid	T1	N0	G1	I	66
16	Endometrioid	T1a	NX	G1	IA	62
17	Endometrioid	T1a	NX	G2	IA	55

Supplementary Figure S1: Hysteroscopy images and Hematoxylin and Eosin (H&E) stained histological sections of representative endometrial biopsies. **(A)** Hysteroscopy (upper panels) and H&E (lower panel) images of representative endometrial biopsies showing Normal, Hyperplastic and Tumoral tissues, respectively. **(B)** Patient and tumor information.

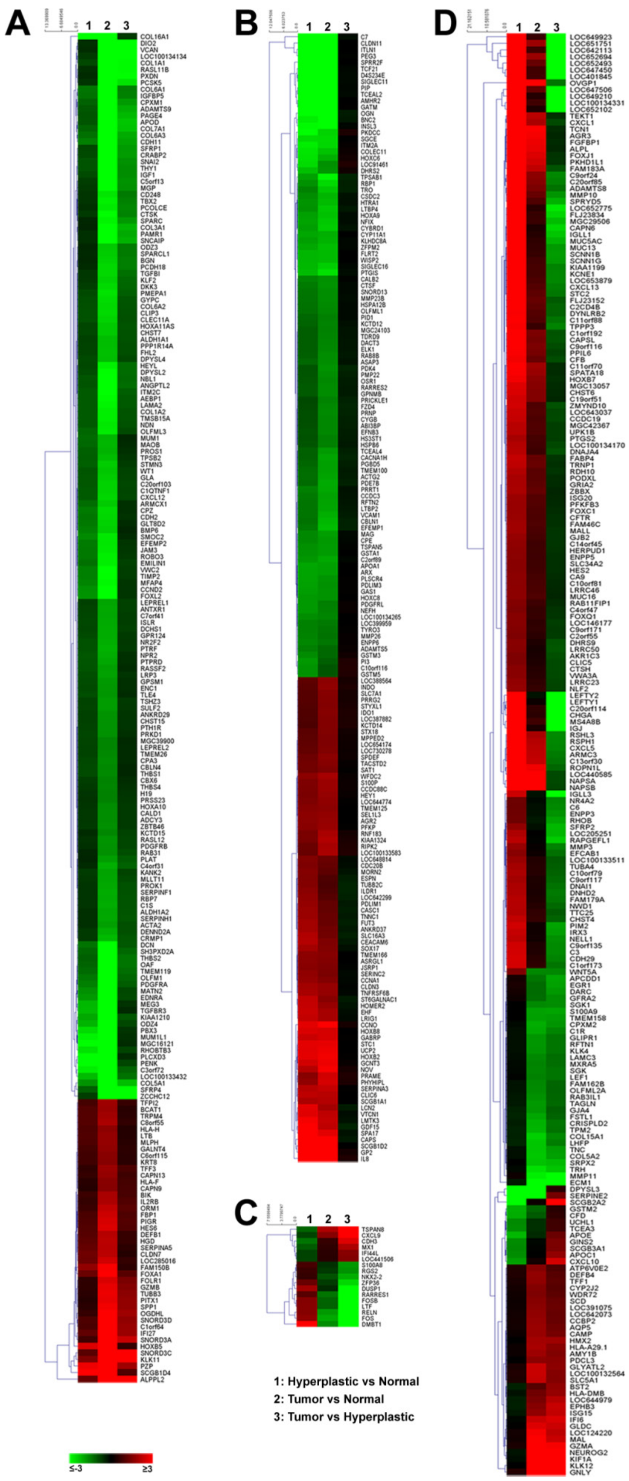

Supplementary Figure S2: Effect of endometrial cell transformation on the transcritptome. Heatmaps showing 618 differentially expressed transcripts (|FC| ≥2.0, *p*Value ≤ 0.001, DiffScore cutoff = 30) in Hyperplastic *vs* Normal (1), Tumoral *vs* Normal (2) and/or Tumoral *vs* Hyperplastic (3) biopsies. Transcripts are grouped as follows: **(A)** those (205) showing a similar pattern of expression changes in the three comparisons considered, **(B)** those (117) found differentially expressed in hyperplasia and not changing further in tumors, **(C)** those (17) showing opposite behavior in hyperplasia and tumors respect to normal and **(D)** those (219) showing different trends in the two pathological tissues.

A

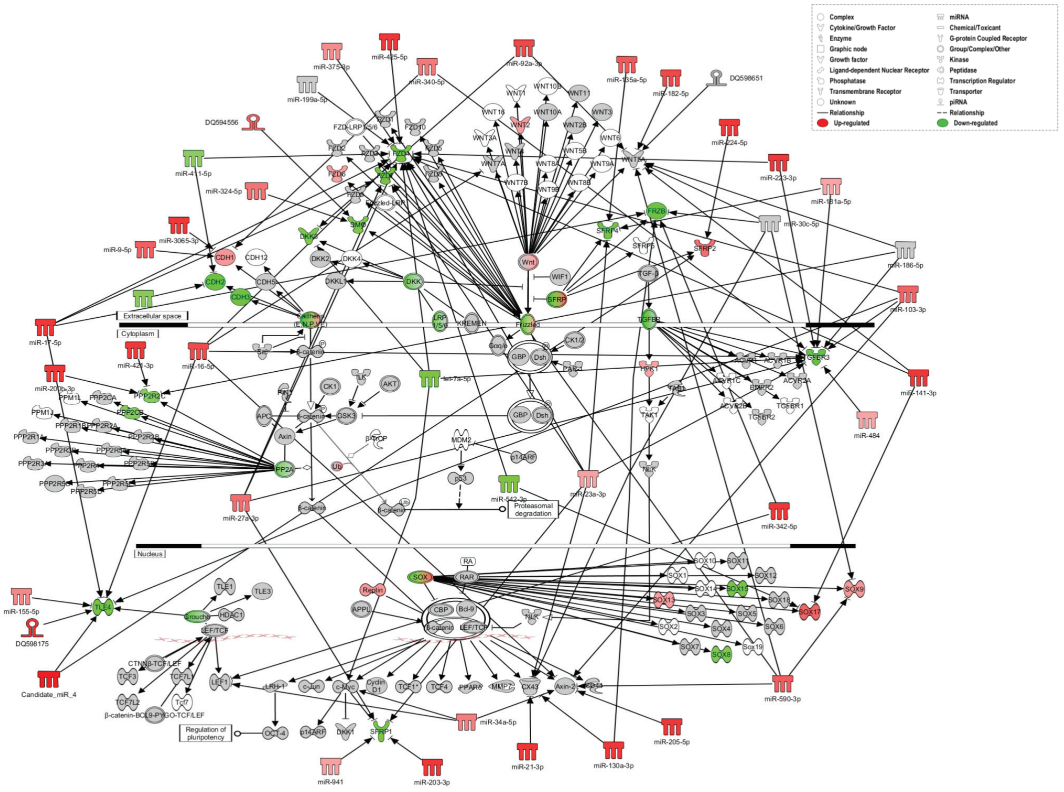

B

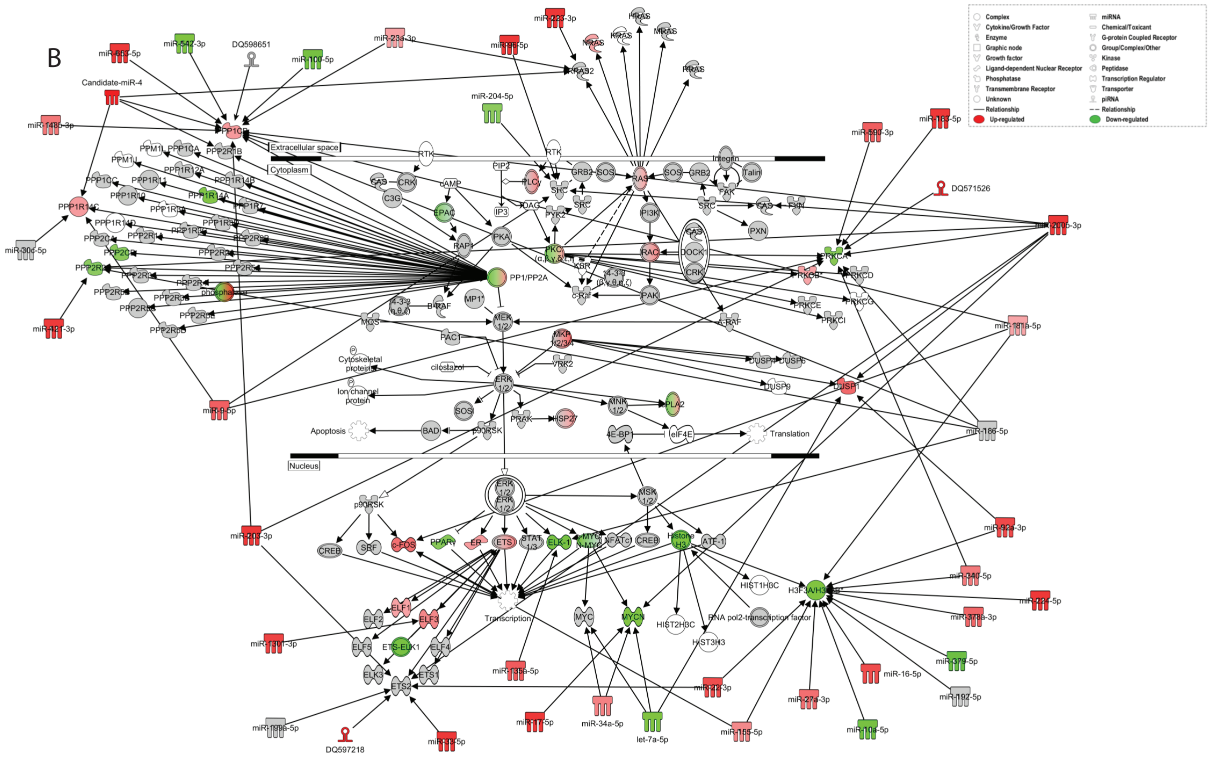

Supplementary Figure S3: Ingenuity pathways analysis. IPA of sncRNA signature and downstream mRNA targets involved in Wnt/β-catenin **(A)**, ERK/MAPK **(B, C)** and TGF-β Signaling **(D, E)** in tumor **(C, E)** and hyperplastic **(A, B, D)** samples, compared with normal endometrium from the same patients. sncRNAs and mRNAs with significant increase or decrease in expression in pathological tissues are shown in red and green, respectively. Those detected but not showing significant differences in expression are shown in gray. Color intensity reflects level of expression. (*Continued*)

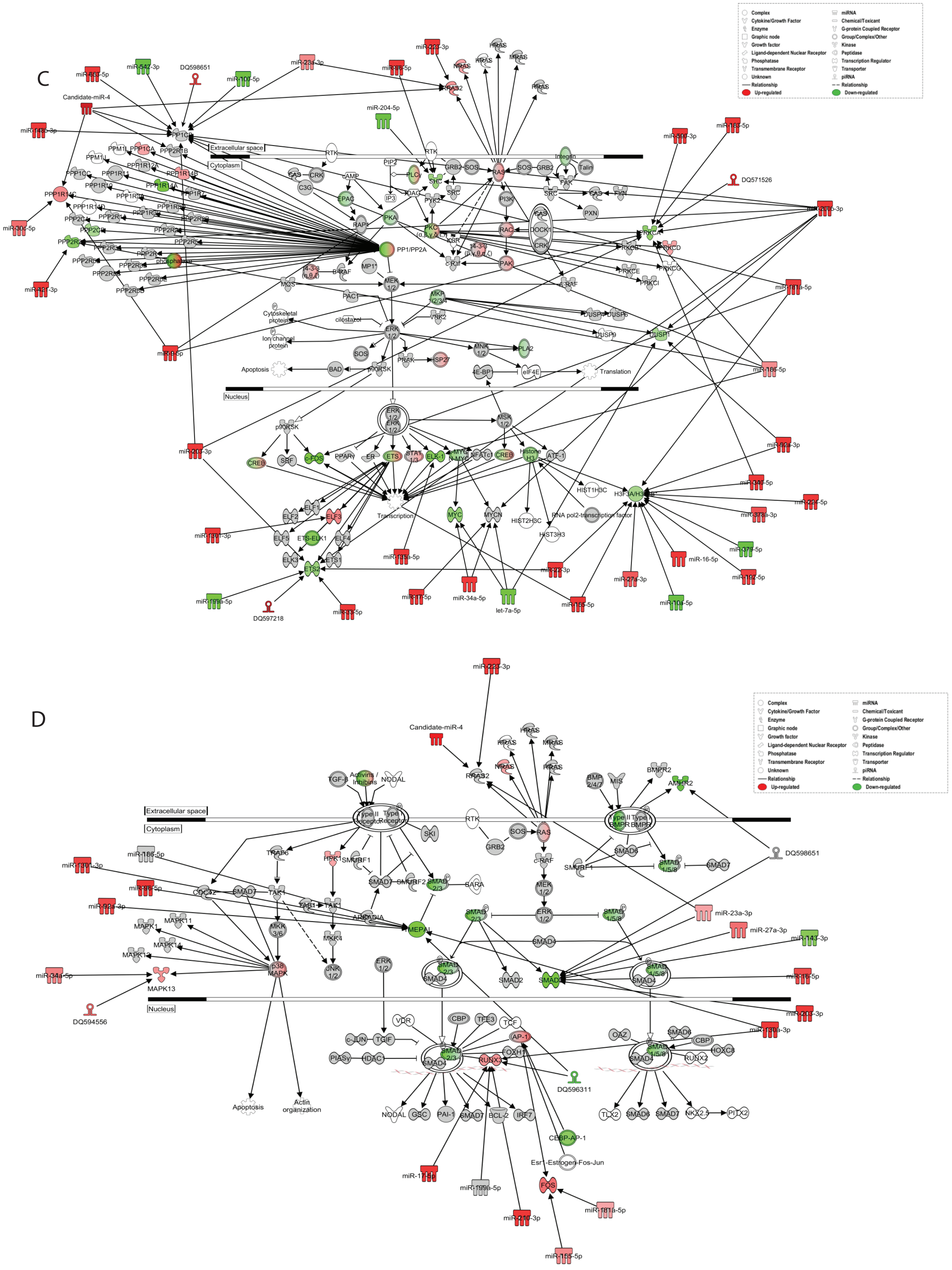

Supplementary Figure S3: (*Continued*) Ingenuity pathways analysis. IPA of sncRNA signature and downstream mRNA targets involved in Wnt/β-catenin **(A)**, ERK/MAPK **(B, C)** and TGF-β Signaling **(D, E)** in tumor **(C, E)** and hyperplastic **(A, B, D)** samples, compared with normal endometrium from the same patients. sncRNAs and mRNAs with significant increase or decrease in expression in pathological tissues are shown in red and green, respectively. Those detected but not showing significant differences in expression are shown in gray. Color intensity reflects level of expression. (*Continued*)

E

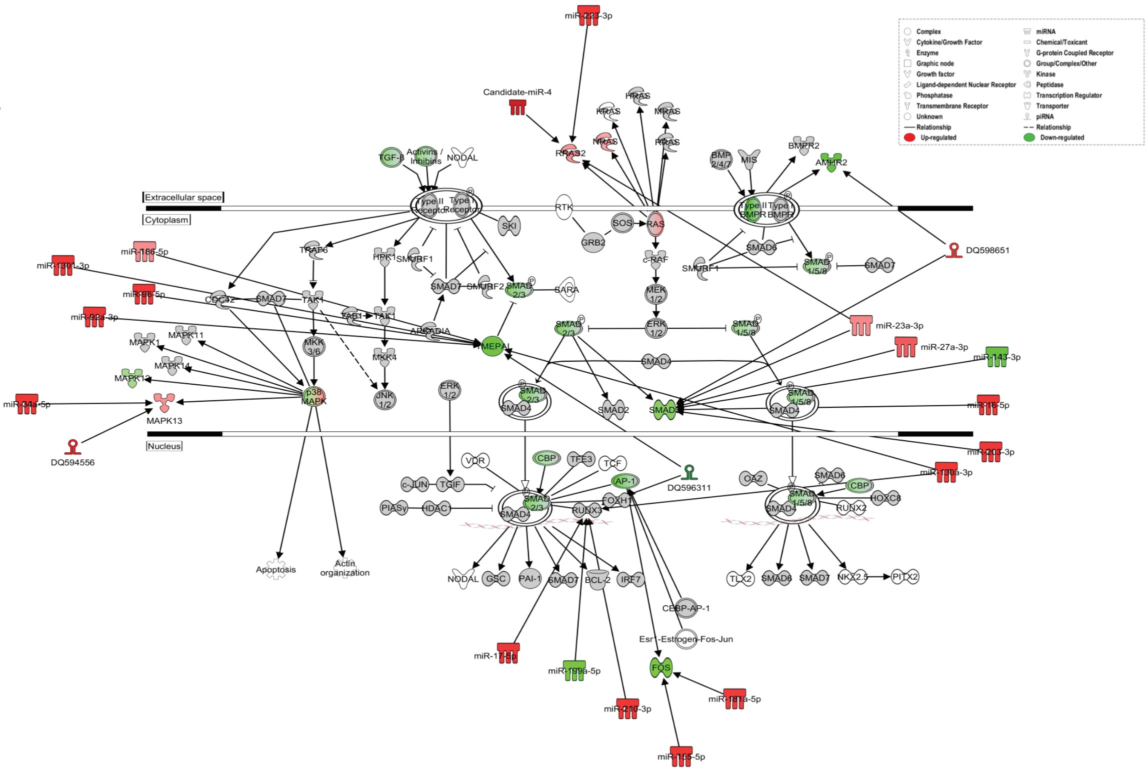

Supplementary Figure S3: Ingenuity pathways analysis. IPA of sncRNA signature and downstream mRNA targets involved in Wnt/β-catenin **(A)**, ERK/MAPK **(B, C)** and TGF-β Signaling **(D, E)** in tumor **(C, E)** and hyperplastic **(A, B, D)** samples, compared with normal endometrium from the same patients. sncRNAs and mRNAs with significant increase or decrease in expression in pathological tissues are shown in red and green, respectively. Those detected but not showing significant differences in expression are shown in gray. Color intensity reflects level of expression.

Supplementary Table 1: The 1268 sncRNAs identified by next generation sequencing in all endometrial tissue samples (N = Normal tissue, H = Hyperplastic tissue & T = Tumor tissue). Reads from technical replicates were summed and normalized using reads per million method (RPM). sncRNAs supported by more than 10 RPM in at least three tissue samples were considered as expressed

Supplementary Table 2: sncRNAs identified to be altered in hyperplastic and/or tumor tissues compared with normal tissue using Bioconductor DESeq package. The file includes fold-change value and the *p*-value associated to each sncRNA. In bold are reported fold-changes with significant *p*-value (≤0.05); N = Normal Tissue, H = Hyperplastic tissue & T = Tumor tissue

Supplementary Table 3: Seven novel miRNAs identified and altered in hyperplastic and/or tumor tissues compared with normal tissue using Bioconductor DESeq package. The file includes miRNAs characteristics, total number of reads identified, fold-change value and the p-value associated to each candidate miRNA. In bold are reported fold-changes with significant p-value (≤0.05).

Supplementary Table 4: The 142 sncRNAs signature identified by next generation sequencing in all endometrial tissue samples. In bold are reported fold-changes with significant *p*-value (≤0.05); N = Normal Tissue, H = Hyperplastic tissue & T = Tumor tissue

Supplementary Table 5: mRNAs identified to be altered in hyperplastic and/or tumor tissues compared with normal tissue by microarrays. The file includes all charactheristics, fold-change value and the *p*-value associated to each mRNA. In bold are reported fold-changes with significant diff. *p*-value (≤0.001)

Supplementary Table 6: Functional annotation analysis of canonical pathways involving the mRNAs target of new miRNAs identified in th signature. For each canonical pathways, the range of statistically significant *p*-values (-log(*p*-value)) and the mRNAs involved are shown

Supplementary Table 7: Functional annotation analysis of canonical pathways involving the mRNAs target of piRNAs identified in the signature. For each canonical pathways, the range of statistically significant *p*-values (-log(*p*-value)) and the mRNAs involved are shown

Supplementary Table 8: 127 known miRNAs identified in the signature, grouped in 79 miRNA families (sharing the same seed region) and the corresponding 8418 putative mRNA target. 909 mRNA target with |FC|≥ 1.5 and *p*-value 0.001 in hyperplastic and/or tumor tissues were considered as differentially expressed (Bold). The file includes miRNAs and target mRNAs fold-change values and Ingenuity Canonical Pathways. N = Normal Tissue, H = Hyperplastic tissue & T = Tumor tissue

Supplementary Table 9: Functional annotation analysis of canonical pathways involving the mRNAs target of 127 miRNAs identified in th signature. For each canonical pathways, the range of statistically significant *p*-values (-log(*p*-value)) and the mRNAs involved are shown
